# Absence of diabetic retinopathy in a patient who has had diabetes mellitus for 69 years, and inadequate glycemic control: case presentation: response

**DOI:** 10.1186/1758-5996-2-20

**Published:** 2010-03-25

**Authors:** Rajiv Raman, Aditi Gupta

**Affiliations:** 1 Shri Bhagwan Mahavir Vitreoretinal Services, 18, College Road, Sankara Nethralaya, Chennai-600 006, Tamil Nadu, India

## Abstract

A response to Jorge Esteves, Carolina Maurente da Rosa, Caroline Kaercher Kramer, Luiz Eduardo Osowski, Stéfano Milano and Luís Henrique Canani: Absence of diabetic retinopathy in a patient who has had diabetes mellitus for 69 years, and inadequate glycemic control: case presentation. Diabetology & Metabolic syndrome 2009, 1:13.

## Response

We read with interest the case described by Jorge Esteves et al. It is a well-documented case of a 73-year-old woman who had type 1 diabetes mellitus for the past 69 years, with no evidence of diabetic retinopathy (DR), despite poor glycemic control and several risk factors for DR including the long duration of the disease, early age at diagnosis of type 1 diabetes mellitus and presence of hypertension and cardiovascular disease.

The patient's case was regularly followed in the Department of Ophthalmology for the past 20 years. Recently, there were many reports of spontaneous regression of DR [1-3]. It could be possible that the patient also developed DR in the initial stages or during the course of the disease which later regressed spontaneously. On visits for ophthalmic evaluation, the retinal examination might have been normal with no signs of spontaneously regressed DR.

There are certain documented ocular and systemic factors which are known to protect a diabetic patient against the development of DR. These include high myopia, raised intraocular pressure and moderate carotid stenosis. The patient's intraocular pressure was always within normal range but there was no mention of her refractive status. If she was a high myope, this could have contributed to protection from DR. Also, since she was known to have significant macroangiopathy manifested by peripheral vasculopathy and cardiovascular disease (myocardial infarction), a carotid doppler examination could be planned at her next follow-up to rule out carotid insufficiency as a factor providing her protection against development of DR.

The authors have also drawn attention to the existence of locally protective genetic factors. They have referred to some studies on the genetics of DR in which the genes of aldose reductase, RAGE, VEGF, ACE, NOS, ICAM-1 and PPAR-d have been considered candidates for DR. We have also identified genetic factors implicated in the predisposition to DR or protection against the development of DR [4-9]. RAGE, the receptor for AGE, gains its importance in the disease mechanism of DR through its interaction with AGE and subsequent initiation of a series of intracellular changes. While analysis of polymorphisms in RAGE gene yielded no association with retinopathy in other studies, we reported a protective association of Gly82Ser for development of DR [5]. Later we found that the Z-2 allele of aldose reductase gene was associated with risk for DR among the Indian population, comparable to worldwide results [6]. We also investigated a promoter microsatellite (GT)n in the *TNF *gene for association with retinopathy in a self reported diabetic cohort. We found a low risk allele ((GT)9) and a high risk allele ((GT)13) for DR in this gene [7]. Later we worked on a pentanucleotide repeat poly-morphism (CCTTT)n in inducible NOS (iNOS) gene. We found a moderate association of two low risk alleles ((CCTTT)13&17) and a high risk allele ((CCTTT)13) for DR [8]. Recently, we also reported that the 27 bp intron4 VNTR of eNOS gene was not associated with DR in a population-based South Indian cohort, much to the contradiction of worldwide reports [9].

Although there seems to be some biologically plausible mechanisms which offer some protection against DR, a study of a larger cohort of such subjects with diabetes, including systemic, local and genetic factors will provide better evidences.

## Competing interests

The authors declare that they have no competing interests.

## Authors' contributions

Authors RR and AG read the article, analyzed it and wrote the manuscript. Both authors read and approved the final manuscript.
